# Manifestations cardiaques de la maladie de Takayasu: à propos d'une observation et revue de la literature

**DOI:** 10.11604/pamj.2016.24.82.9320

**Published:** 2016-05-25

**Authors:** Abdelmajid Bouzerda, Ali khatouri

**Affiliations:** 1 Service de Cardiologie, 1 Centre Médico-chirurgical, Agadir, Maroc; 2 Service de Cardiologie, Hôpital Militaire Avicenne, Université Cadi Ayyad, Faculté de Médecine et Pharmacie, Marrakech, Maroc

**Keywords:** Takayasu´s arteritis, coronaritis, review, Takayasu's disease, coronaritis, review

## Abstract

La maladie de Takayasu est une maladie vasculaire inflammatoire rare, touchant préférentiellement la jeune femme avec une atteinte prépondérante de l'aorte et ses premières branches de divisions. Nous rapportons le cas d'une atteinte ostiale du tronc commun gauche au décours d'un syndrome coronarien aigu ST négatif révélant une maladie de Takayasu et nous détaillerons les différentes manifestations cardiovasculaires de cette maladie.

## Abstract (en)

Takayasu's disease is a rare inflammatory vascular disease, preferentially affecting young woman with predominant involvement of the aorta and its first division branches. We here report the case of a patient with ostial left main trunk involvement following non-ST elevation acute coronary syndrome revealing Takayasu's disease and we highlight the various cardiovascular manifestations of this disease.

## Introduction

La maladie de Takayasu est une artérite inflammatoire chronique, d’étiologie inconnue touchant préférentiellement la femme jeune, avec une atteinte segmentaire de l'aorte et de ses branches principales. L’épaississement de la paroi vasculaire est le signe précoce le plus caractéristique de la maladie, aboutissant progressivement a des sténoses, des thromboses et parfois au développement d'anévrismes. Nous rapportons le cas d'une atteinte ostiale du tronc commun de la coronaire gauche révélant une maladie de Takayasu.

## Patient et observation

**Figure 1:**
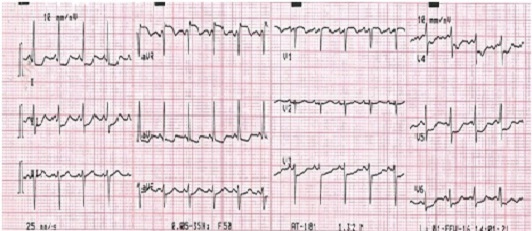
Sous décalage ST en antérieur étendu, en inférieur avec sus décalage en Avr

**Figure 2:**
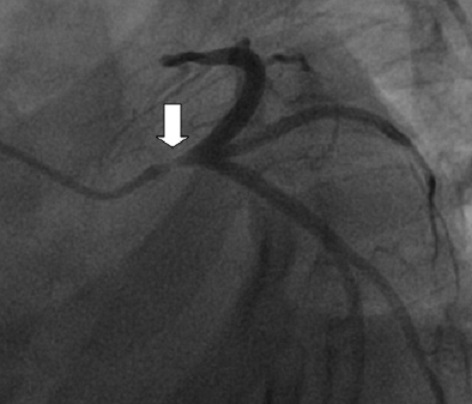
Incidence OAG caudale montrant une sténose subocclusive de l'ostium du tronc commun de l'artère coronaire gauche (flèche)

**Figure 3:**
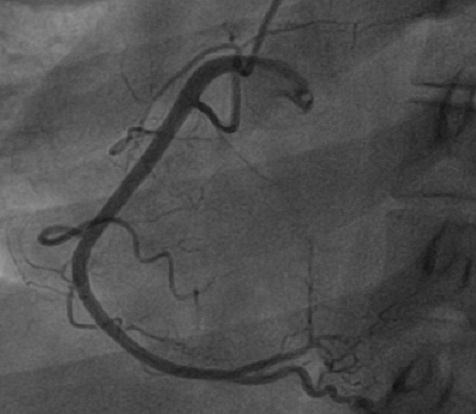
Incidence OAG montrant un réseau coronaire droit indemne de lésions

A.S jeune femme de 34 ans, sans antécédents médicaux particuliers ni facteurs de risque cardiovasculaires. Admise pour des douleurs thoraciques angineuses intenses, rétrosternale constrictive, irradiant vers le membre supérieur gauche et le maxillaire inférieur, associées à des vomissements et des sueurs profuses. L'examen clinique à son admission note une patiente qui souffre au repos, ses conjonctives sont normalement colorées, tachycarde à 100bpm avec une TA à 110/75 mmhg L'examen cardiaque trouve des bruits du cœur réguliers, avec un bruit de galop gauche. Les pouls périphériques sont présents de manière bilatérale et symétrique sans souffle à l'auscultation des gros axes vasculaires accessibles. L'examen pleuropulmonaire note des crépitants des deux bases sans signes périphériques d'insuffisance cardiaque droite. Le reste de l'examen somatique est sans anomalies. L’électrocardiogramme basal de repos inscrit un RRS à 150 c/min, un sous décalage du segment ST en antérieur étendu et en inférieurs avec un sus décalage ST en AVr (Figure 1) Le bilan biologique montre des troponines I à 100 fois la normale. L’échocardiographie transthoracique de repos montre un VG dilaté, une hypocinésie globale asymétrique avec une fonction systolique ventriculaire gauche altérée (FE estimée à 25% par le Simpson biplan). Des pressions de remplissage élevées. Par ailleurs, il n'y a pas d´épanchement péricardique ni thrombus intracavitaire ni anomalies valvulaires. Le diagnostic de Syndrome coronaire aigu ST négatif à haut risque est retenu et Un traitement pharmacologique à base de Clopidogrel (Plavix*) 600 mg, Enoxaparine (Lovenox*) IV 0.5mg /kg, Aspégic 250 mg en IV, Morphine, Furosémide (Lasilix*) 40 mg est instauré aux urgences, puis adressé en unité de cardiologie interventionnelle pour coronarographie. La coronarographique montre une sténose subocclusive isolée de l'ostium du tronc commun de l'artère coronaire gauche (Figure 2). Le réseau droit est indemne de lésions (Figure 3). La patiente a bénéficié en urgence d'un pontage aorto-coronaire avec des suites post opératoire simples. L'examen anatomopathologique de la biopsie de l'artère mammaire interne montre des lésions évocatrices de la maladie de Takayasu. L'Echodoppler des troncs supra-aortiques a mis en évidence un épaississement pariétal au niveau de l'artère carotide interne droite et de la sous clavière dont le caractère est homogène et circonférentiel confirmant la maladie de Takayasu. Le patient quitte le service sous traitement médical suivant: clopidogrel (Plavix^®^ 75 mg/j), aspirine (Kardégic^®^ 75 mg/j), bêtabloquant (Sectral^®^ 200 mg/j), IEC (Triatec^®^ 5 mg/j) et rosuvastatine (Crestor^®^ 20 mg/j), prednisone 0.5mg/kg puis adressée au service de Médecine interne pour suivi.

## Discussion

La maladie de Takayasu est une artérite inflammatoire chronique d'origine inconnue qui affecte les vaisseaux de gros calibre principalement l'aorte et ses branches principales. La prévalence de la maladie est plus importante au Japon (40 par millions d'habitants), en Amérique latine et en Afrique avec une incidence annuelle entre 2 à 3 cas par millions d'habitants. Il s'agit d'une maladie de sujet jeune survenant au cours des 2 ou 3 décennies avec une prédominance féminine (62% à 97% des patients selon les études). La cause de la maladie demeure inconnue, quelques cas d'atteinte familiale ont été décrits, une étude [1] retrouve les gènes codant pour les interleukines 12B, IL 2 et IL 6 comme loci de susceptibilité. Il existe dans la plupart des séries une prévalence élevée de tuberculoses avérées [2], enfin la réponse immunitaire Th1 et Th 17 semble jouer un rôle important dans l'activité de la maladie [3]. Sur le plan clinique il est classique de distinguer la période aigue dite préocclusive de la phase occlusive caractérisée par des manifestations ischémiques. La période préocclusive ou phase systémique associe des signes généraux, des signes cutanés (érythème noueux, pyoderma gangrenosum), des douleurs sur les trajets artériel et parfois une atteinte ophtalmologique: épisclérite, uvéite antérieure. La période occlusive ou phase vasculaire est la conséquence des lésions artérielles (sténose, oblitération, anévrisme). L'atteinte cardiaque est retrouvée dans 30 à 40% des cas, considérée comme l'un des critères de sévérité de la maladie [4]. La fréquence de l'atteinte coronarienne est variable selon les séries (5 à 45%) elle résulte d'une atteinte ostiale (le cas de notre observation) ou proximale et se manifeste le plus souvent par un angor, cette atteinte est souvent associée a une aortite et une atteinte des artère sous clavière et pose parfois un problème thérapeutique en terme de revascularisation [5]. L'atteinte myocardique clinique est rare mais des anomalies de perfusion sont fréquemment observée en scintigraphie au thallium (84%) et des rehaussements tardifs au gadolinium en IRM (26%) sans atteinte coronaire [6]. La fuite aortique a été rapportée dans 13 à 25% des cas [7], secondaire soit à une dilatation annulaire suite à un anévrisme de l'aorte ascendante, soit par rétraction des cuspides aortiques, mais l'association des deux mécanismes reste la plus fréquente. Le remplacement valvulaire aortique (RVA) est la seule alternative thérapeutique pour corriger la fuite aortique au cours de cette maladie. Il va améliorer la fonction ventriculaire gauche [8], en revanche certaines complications peuvent être observées en postopératoire et nécessitant une reprise chirurgicale, notamment les désinsertions prothétiques, les pseudo anévrismes et les endocardites sur prothèse dues à la fragilité tissulaire et à l'inflammation. L'hypertension artérielle est très fréquente dans la maladie de Takayasu elle peut être due à diverses étiologies: atteinte artérielle rénale, pseudo coarctation aortique et rigidité pariétale secondaire à l'atteinte vasculaire, élargissement de la différentielle en cas d'insuffisance aortique. Une hypertension artérielle maligne doit faire rechercher une sténose bilatérale des artères rénales. La claudication intermittente des membres inférieurs peut révéler la coexistence de sténoses et de dilatations ou anévrisme de l´aorte thoracique ou l'aorte abdominale, très évocatrices de la maladie surtout lorsque la paroi vasculaire est épaissie. L'atteinte des vaisseaux digestifs (tronc cœliaque et artères mésentériques, est assez fréquente, mais la survenue d'un angor mésentérique est rare. Enfin, les sténoses des artères rénales sont fréquentes et responsable d'une hypertension rénovasculaire. Le diagnostic est fondé sur l'imagerie. Actuellement, l’écho-Doppler, l'angio- tomodensitométrie et l'imagerie par résonance magnétique nucléaire constituent des méthodes fiables et rapides d’évaluation de la lumière mais aussi de la paroi des vaisseaux. La qualité de vie est fortement altérée au cours de la maladie de Takayasu, alors que le pronostic est généralement bon. Les principales causes de décès sont l'insuffisance cardiaque, la survenue d'accidents vasculaires cérébraux, l'insuffisance rénale et la persistance d'un syndrome inflammatoire. L'angioplastie percutanée transluminale et parfois la chirurgie de revascularisation sont nécessaire en cas d'ischémie critique. La corticothérapie est le traitement de première ligne, en cas d’échec l'adjonction de methothrexate permettrait de contrôler la maladie.

## Conclusion

La fréquence des atteintes cardiaques au cours de la maladie de Takayasu est probablement sous-estimée dans la littérature. Les atteintes les plus fréquentes sont valvulaires aortiques et coronaires et leur survenue conditionne en partie le pronostic de la maladie.
